# Supplementary material for: Development and characterization of an antibody that recognizes influenza virus N1 neuraminidases
Source: PLoS One. 2024 May 9;19(5):e0302865. doi: 10.1371/journal.pone.0302865 (PMC11081314; doi:10.1371/journal.pone.0302865)
Supplement: S1 File — (DOCX) [file pone.0302865.s001.docx]

**Raw Data for figure 1 – 3, excluding the data of flow cytometry experiments**

**Data for figure 1**

**(a)** phage ELISA

| 1 | 2 | 3 | 4 | 5 | 6 | 7 | 8 | 9 | 10 | 11 | 12 |
| --- | --- | --- | --- | --- | --- | --- | --- | --- | --- | --- | --- |
| 0.7409 | 0.4285 | 1.1296 | 0.8023 | 0.844 | 0.8379 | 0.3628 | 0.5395 | 0.389 | 0.4286 | 0.5501 | 0.5608 |
| 0.1739 | 0.8213 | 0.6332 | 0.2634 | 0.7939 | 1.0158 | 0.4497 | 0.3333 | 0.2695 | 0.2604 | 0.2029 | 0.1825 |
| 0.0656 | 0.4106 | 0.3772 | 0.953 | 0.1127 | 0.7639 | 0.0541 | 0.4904 | 0.0555 | 0.3342 | 0.2462 | 0.3177 |
| 0.079 | 0.364 | 0.4493 | 0.0649 | 0.6233 | 0.2613 | 0.8042 | 0.7809 | 0.2714 | 0.084 | 0.8307 | 0.5037 |
| 0.4549 | 0.2403 | 1.0251 | 0.7696 | 0.7359 | 0.0566 | 0.0651 | 0.3677 | 0.1234 | 0.1664 | 0.3973 | 0.3907 |
| 0.0826 | 0.7319 | 0.0632 | 0.0701 | 0.0804 | 0.0629 | 0.2615 | 1.3061 | 0.0767 | 0.3264 | 0.549 | 0.0705 |
| 0.5584 | 0.2251 | 0.165 | 0.2147 | 0.0544 | 0.0663 | 0.2189 | 0.4377 | 0.2153 | 0.5743 | 0.4621 | 0.8383 |
| 0.0743 | 0.0579 | 0.0521 | 0.0539 | 0.0494 | 0.0522 | 0.0488 | 0.0501 | 0.9229 | 0.8683 | 1.1584 | 0.8051 |
| negative controls | | | | | | | |  |  |  |  |

**(b)** SDS-PAGE analysis

**
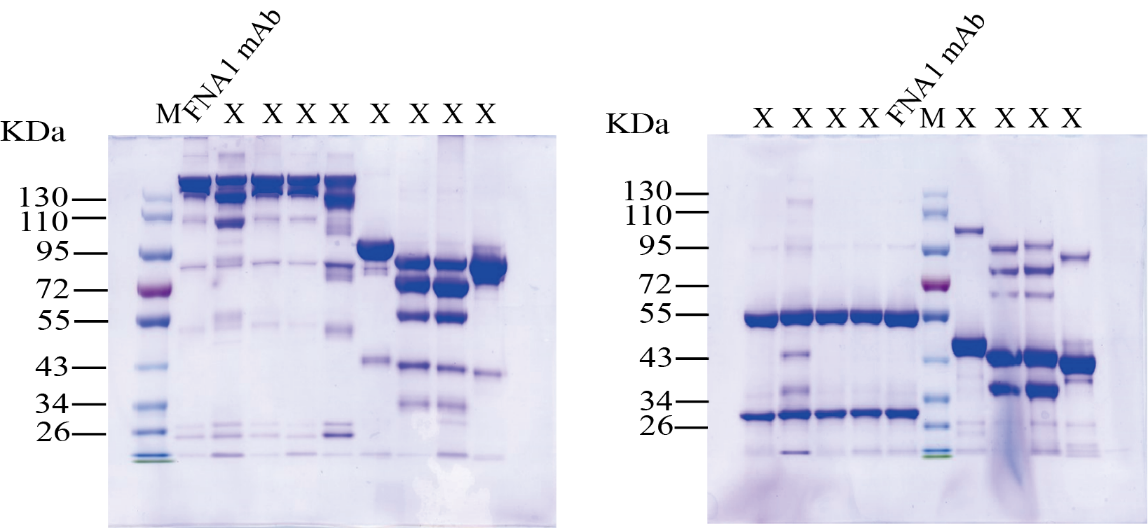
**

**Data for Figure 2**

**(a)** ELISA

| FNA1 mAb concentration | H1N1 NA | H3N2 NA | H5N1 NA | H7N9 NA |
| --- | --- | --- | --- | --- |
| 5 μg/mL | 3.3819 | 0.0396 | 0.7413 | 0.0397 |
| 2.5 μg/mL | 3.0203 | 0.0543 | 0.5877 | 0.0625 |
| 1.25 μg/mL | 1.9769 | 0.0413 | 0.2879 | 0.0417 |
| 0.625 μg/mL | 1.2159 | 0.047 | 0.1889 | 0.0506 |
| 0.3125 μg/mL | 0.7709 | 0.0444 | 0.1027 | 0.0451 |
| 0.15625 μg/mL | 0.3386 | 0.0425 | 0.0709 | 0.0421 |
| 0.078125 μg/mL | 0.2655 | 0.0393 | 0.0535 | 0.0367 |
| 0 μg/mL | 0.0397 | 0.0478 | 0.0425 | 0.0407 |

**(b)** The antigen-binding activities of FNA1 mAb to the NAs of H1N1, H3N2, H5N1, and H7N9 were assessed by western blot.


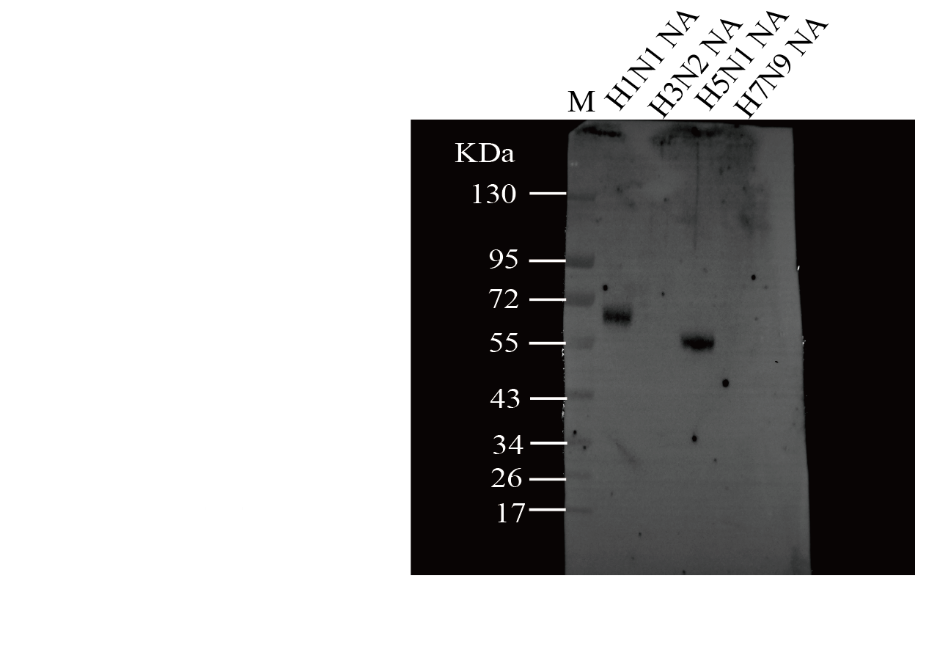


**(d)**

Affinity determination of FNA1 mAb to CA/09 NA

| Sensor Location | Sensor Type | Sample ID | Conc. (nM) | KD (M) | kon(1/Ms) | kon Error | kdis(1/s) | kdis Error | Full R^2 |
| --- | --- | --- | --- | --- | --- | --- | --- | --- | --- |
| A1 | Ni-NTA | FNA1 | 666.7 | 7.82E-08 | 1.72E+04 | 2.56E+02 | 1.35E-03 | 1.85E-05 | 0.981201 |
| B1 | Ni-NTA | FNA1 | 333.3 | 7.82E-08 | 1.72E+04 | 2.56E+02 | 1.35E-03 | 1.85E-05 | 0.981201 |
| C1 | Ni-NTA | FNA1 | 166.7 | 7.82E-08 | 1.72E+04 | 2.56E+02 | 1.35E-03 | 1.85E-05 | 0.981201 |
| D1 | Ni-NTA | FNA1 | 83.3 | 7.82E-08 | 1.72E+04 | 2.56E+02 | 1.35E-03 | 1.85E-05 | 0.981201 |
| E1 | Ni-NTA | FNA1 | 41.7 | 7.82E-08 | 1.72E+04 | 2.56E+02 | 1.35E-03 | 1.85E-05 | 0.981201 |

Affinity determination of FNA1 mAb to Anh05 NA

| Sensor Location | Sensor Type | Sample ID | Conc. (nM) | KD (M) | kon(1/Ms) | kon Error | kdis(1/s) | kdis Error | Full R^2 |
| --- | --- | --- | --- | --- | --- | --- | --- | --- | --- |
| A2 | Ni-NTA | FNA1 | 666.7 | 2.97E-08 | 1.20E+04 | 1.19E+02 | 3.55E-04 | 4.59E-06 | 0.992371 |
| B2 | Ni-NTA | FNA1 | 333.3 | 2.97E-08 | 1.20E+04 | 1.19E+02 | 3.55E-04 | 4.59E-06 | 0.992371 |
| C2 | Ni-NTA | FNA1 | 166.7 | 2.97E-08 | 1.20E+04 | 1.19E+02 | 3.55E-04 | 4.59E-06 | 0.992371 |
| D2 | Ni-NTA | FNA1 | 83.3 | 2.97E-08 | 1.20E+04 | 1.19E+02 | 3.55E-04 | 4.59E-06 | 0.992371 |
| E2 | Ni-NTA | FNA1 | 41.7 | 2.97E-08 | 1.20E+04 | 1.19E+02 | 3.55E-04 | 4.59E-06 | 0.992371 |

**Data for Figure 3**

**(a)** ELLA

| mAb concentration | FNA1 | WN1(an irrelevant antibody) | negative controls |
| --- | --- | --- | --- |
| 100 μg/mL | 0.221 | 1.479 | 0.102 |
| 20 μg/mL | 0.267 | 1.376 | 0.108 |
| 4 μg/mL | 0.4 | 1.76 | 0.11 |
| 0.8 μg/mL | 0.814 |  | 0.114 |
| 0.16 μg/mL | 1.221 | 1.787 |  |
| 0.032 μg/mL | 1.305 | 1.68 |  |
| 0.0064 μg/mL | 1.412 | 1.505 |  |
| 0 μg/mL | 1.283 | 1.549 |  |

**(b)** ELLA

| FNA1 mAb concentration | CA/09 NA | Anh05 NA | negative controls |
| --- | --- | --- | --- |
| 100 μg/mL | 0.405 | 0.255 | 0.127 |
| 20 μg/mL | 0.337 | 0.303 | 0.136 |
| 4 μg/mL | 0.329 | 1.107 | 0.096 |
| 0.8 μg/mL | 0.367 | 2.332 |  |
| 0.16 μg/mL | 0.493 | 2.56 |  |
| 0.032 μg/mL | 0.952 | 2.62 |  |
| 0.0064 μg/mL | 1.301 | 2.628 |  |
| 0 μg/mL | 1.131 | 2.6 |  |

**(c)** In vitro inhibition of pseudovirus release by FNA1 mAb

| Control | FNA1 5 μg/ml | FNA1 50 μg/ml |
| --- | --- | --- |
| 2.80E+05 | 3.59E+04 | 1.06E+04 |
| 2.43E+05 | 3.35E+04 | 9.85E+03 |

**(d)**  Neutralization test of the FNA1 mAb in vitro

| 1.82E+04 | FHA3 5ug/ml | 1.14E+06 | FNA1 5ug/ml | 1.17E+06 | wells without antibody |
| --- | --- | --- | --- | --- | --- |
| 1.70E+04 |  | 1.30E+06 |  | 1.47E+06 |  |
| 1.77E+04 |  | 1.36E+06 |  | 1.42E+06 |  |
| 1.10E+05 |  | 1.48E+06 |  | 1.43E+06 |  |
| 8.21E+03 | FHA3 50ug/ml | 1.50E+06 | FNA1 50ug/ml |  |  |
| 7.03E+03 |  | 1.62E+06 |  |  |  |
| 2.70E+03 |  | 1.52E+06 |  |  |  |
| 1.40E+03 |  | 1.57E+06 |  |  |  |
