# Supplementary figures and images for: Development and characterization of an antibody that recognizes influenza virus N1 neuraminidases
Source: PLoS One. 2024 May 9;19(5):e0302865. doi: 10.1371/journal.pone.0302865 (PMC11081314; doi:10.1371/journal.pone.0302865)

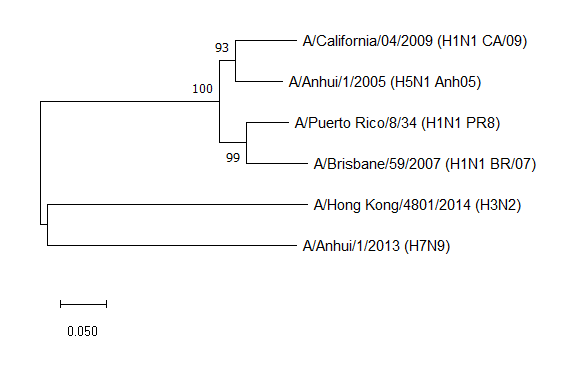

Supplement: S1 Fig — (PNG) [file pone.0302865.s002.png]

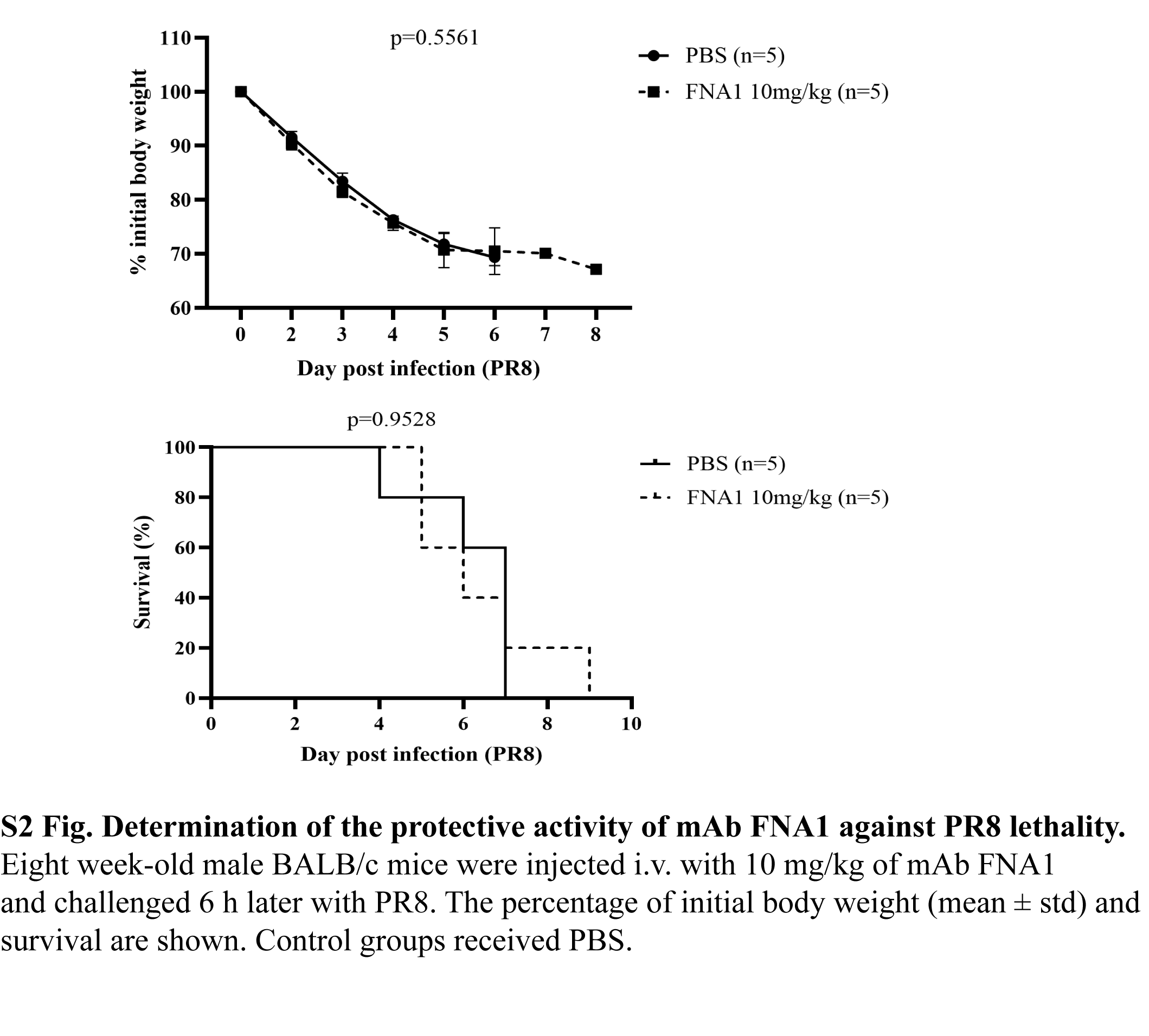

Supplement: S2 Fig — (TIF) [file pone.0302865.s003.tif]
