# Supplementary material for: Development and characterization of an antibody that recognizes influenza virus N1 neuraminidases
Source: PLoS One. 2024 May 9;19(5):e0302865. doi: 10.1371/journal.pone.0302865 (PMC11081314; doi:10.1371/journal.pone.0302865)
Supplement: Attachment — Submitted filename: Response to Reviewers(2).docx [file pone.0302865.s008.docx]

**Response to academic editor and reviewer**

**PLOS ONE**

**Article Title:** Development and characterization of an antibody recognizing the influenza virus N1 neuraminidases

**Running Number:** PONE-D-23-41925

**Response to academic editor**

*1.* *Please ensure that your manuscript meets PLOS ONE's style requirements, including those for file naming. The PLOS ONE style templates can be found at*

*https://journals.plos.org/plosone/s/file?id=wjVg/PLOSOne_formatting_sample_main_body.pdf and*

*https://journals.plos.org/plosone/s/file?id=ba62/PLOSOne_formatting_sample_title_authors_affiliations.pdf*

**Response:** In accordance with PLOS ONE's style requirements, we have adjusted the formatting of our manuscript entitled “Development and characterization of an antibody recognizing the influenza virus N1 neuraminidases” and followed the file naming process.

*2. PLOS requires an ORCID iD for the corresponding author in Editorial Manager on papers submitted after December 6th, 2016. Please ensure that you have an ORCID iD and that it is validated in Editorial Manager. To do this, go to ‘Update my Information’ (in the upper left-hand corner of the main menu), and click on the Fetch/Validate link next to the ORCID field. This will take you to the ORCID site and allow you to create a new iD or authenticate a pre-existing iD in Editorial Manager. Please see the following video for instructions on linking an ORCID iD to your Editorial Manager account: https://www.youtube.com/watch?v=_xcclfuvtxQ*

**Response:** ORCID iDs of the three corresponding authors of this manuscript have been successfully linked to their Editorial Manager accounts following the provided instructions.

*3. We note that you have included the phrase “data not shown” in your manuscript. Unfortunately, this does not meet our data sharing requirements. PLOS does not permit references to inaccessible data. We require that authors provide all relevant data within the paper, Supporting Information files, or in an acceptable, public repository. Please add a citation to support this phrase or upload the data that corresponds with these findings to a stable repository (such as Figshare or Dryad) and provide and URLs, DOIs, or accession numbers that may be used to access these data. Or, if the data are not a core part of the research being presented in your study, we ask that you remove the phrase that refers to these data.*

**Response:** We acknowledge this suggestion and have added tables in the supporting information (S2 and S3 Tables) to confirm our statement.

*4. Please include captions for your Supporting Information files at the end of your manuscript, and update any in-text citations to match accordingly. Please see our Supporting Information guidelines for more information: http://journals.plos.org/plosone/s/supporting-information.*

**Response:** We have updated the supporting information files by replacing the original ones with a Word file (S1_File.docx), a figure (S1_Fig.png), and three Excel files (S1_Table.xlsx, S2_Table.xlsx, and S3_Table.xlsx). In addition, captions for the supporting information files have been added at the end of our manuscript, following the guidelines for supporting information. We have also updated the in-text citations accordingly (line 292, line 306, and line 330).

**Response to Reviewer #1**

*1. Did the authors test NA subtypes beyond N1, N2 and N7? if not, please explain. Also, a sequence line-up of the NA sequences of the NA used in this study would be helpful for the readerships.*

**Response:** We focused our testing exclusively on NA subtypes N1 (CA/09 NA, PR8 NA, BR/07 NA, and Anh05 NA), N2 (H3N2 NA), and N9 (H7N9 NA). The reason for this is that CA/09 NA was used as the target antigen during phage display selections. Therefore, the antibodies obtained are probably N1-specific. While FNA1 demonstrated binding to CA/09 and Anh05 NA, it exhibited no reactivity with PR8, BR/07, H3N2, and H7N9 NA, which shows its limited binding breadth. Additionally, in response to the reviewer’s recommendation, we aligned the NA amino acid sequences of influenza viruses described in the study, and found that Anh05 NA showed a higher homology between Anh05 NA and CA/09 NA compared to other sequences (line 290–292, S1 Fig and S1 Table).

*2. While this reviewer does like the work, the manuscript, as it stands now, is quite incomplete in terms of being convincingly showcasing the power of the mAb. Clearly, this work could really be elevated to a much higher visibility by conducting protection experiment in mouse model where mAb is shown to demonstrate protection against challenge by H1N1, not H3N2 etc.*

**Response:** We acknowledge the reviewer’s suggestion regarding mouse challenge experiments in terms of demonstrating the protective capacity of the mAb against H1N1, but not other subtypes, e.g., H3N2, which could significantly improve the visibility of our study. Regrettably, we currently lack access to the CA/09 virus. Nevertheless, we carried out an in vivo prophylactic study to evaluate FNA1 mAb's potential in protecting against PR8. Despite our efforts, FNA1 failed to provide protection against PR8, as shown in the subsequent figure.

**Response to Reviewer #2**

*1. Is the antibody screened from phage display the only one binding to N1, or are there others?*

**Response:** Through the use of phage display techniques, we identified three mAbs targeting N1 NAs. While all three mAbs showed binding activity to CA/09 and Anh05 NA, the affinities (KD) of the two other mAbs were lower compared to FNA1. In addition, analysis revealed that these three mAbs shared the same four key residues of H1N1 NA that affected their binding to viral proteins, suggesting that they might target similar sites. Based on these findings, we opted to focus solely on FNA1 for presenting the results.

*2. The author should consider submitting the FNA1 antibody sequence to public databases like NCBI.*

**Response:** We concur with the reviewer’s suggestion regarding the submission of the FNA1 mAb sequence to a public database. Consequently, we have deposited the DNA sequences of both the FNA1 heavy and light chain variable regions at GenBank. The accession numbers for the nucleotide sequences are PP483115 (heavy chain) and PP483116 (light chain).

*3. As a suggestion, the author could use NA protein for ELLA experiments to assess ELLA activity against CA/09 N1.*

**Response:** We agree with the reviewer’s suggestion to utilize NA protein in ELLA experiments to assess the NI activity of FNA1 mAb against CA/09 N1. Accordingly, we conducted ELLA assays using recombinant CA/09 NA and Anh05 NA, and found that FNA1 effectively blocked the activity of both CA/09 NA and Anh05 NA, resulting in the decreased cleavage of fetuin by NA proteins, with IC_50_ values of 0.06 μg/mL and 2.88 μg/mL, respectively (line 306–309 and updated Fig 3b).

*4. In Figure 3c, the author should clarify the nature of FHA3.*

**Response:** We greatly appreciate the reviewer's suggestion and have added a clarifying remark in line 319.
