# Supplementary material for: Development and characterization of an antibody that recognizes influenza virus N1 neuraminidases
Source: PLoS One. 2024 May 9;19(5):e0302865. doi: 10.1371/journal.pone.0302865 (PMC11081314; doi:10.1371/journal.pone.0302865)
Supplement: Attachment — Submitted filename: Response to Reviewers(2).docx [file pone.0302865.s009.docx]

**Response to academic editor and reviewer**

**PLOS ONE**

**Article Title:** Development and characterization of an antibody recognizing the influenza virus N1 neuraminidases

**Running Number:** PONE-D-23-41925R1

**Response to academic editor**

*1.* *Please review your reference list to ensure that it is complete and correct. If you have cited papers that have been retracted, please include the rationale for doing so in the manuscript text, or remove these references and replace them with relevant current references. Any changes to the reference list should be mentioned in the rebuttal letter that accompanies your revised manuscript. If you need to cite a retracted article, indicate the article’s retracted status in the References list and also include a citation and full reference for the retraction notice.*

**Response:** We acknowledge this suggestion and have reviewed the reference list carefully. We discovered that the 30th reference was incomplete, as it lacked the identifier (DOI), and the 31st reference was a manuscript that had just been accepted. Consequently, these two references were removed and replaced with relevant current references (line 499–504).

**Response to Reviewer #1**

*1. Did the authors test NA subtypes beyond N1, N2 and N7? if not, please explain. Also, a sequence line-up of the NA sequences of the NA used in this study would be helpful for the readerships.*

*Response: We focused our testing exclusively on NA subtypes N1 (CA/09 NA, PR8 NA, BR/07 NA, and Anh05 NA), N2 (H3N2 NA), and N9 (H7N9 NA). The reason for this is that CA/09 NA was used as the target antigen during phage display selections. Therefore, the antibodies obtained are probably N1-specific. While FNA1 demonstrated binding to CA/09 and Anh05 NA, it exhibited no reactivity with PR8, BR/07, H3N2, and H7N9 NA, which shows its limited binding breadth. Additionally, in response to the reviewer’s recommendation, we aligned the NA amino acid sequences of influenza viruses described in the study, and found that Anh05 NA showed a higher homology between Anh05 NA and CA/09 NA compared to other sequences (line 290–292, S1 Fig and S1 Table).*

*Reviewer's responses to the latest revised Ms: Understood your limitations. Yet, such limitations should be more elaborated in the Discussion.*

**Response:** We concur with the reviewer’s suggestion regarding the exposition of the limitations of our study in the Discussion, and we have added further relevant content to clarify the limitations (line 370–374).

*2. While this reviewer does like the work, the manuscript, as it stands now, is quite incomplete in terms of being convincingly showcasing the power of the mAb. Clearly, this work could really be elevated to a much higher visibility by conducting protection experiment in mouse model where mAb is shown to demonstrate protection against challenge by H1N1, not H3N2 etc.*

*Response: We acknowledge the reviewer’s suggestion regarding mouse challenge experiments in terms of demonstrating the protective capacity of the mAb against H1N1, but not other subtypes, e.g., H3N2, which could significantly improve the visibility of our study. Regrettably, we currently lack access to the CA/09 virus. Nevertheless, we carried out an in vivo prophylactic study to evaluate FNA1 mAb's potential in protecting against PR8. Despite our efforts, FNA1 failed to provide protection against PR8, as shown in the subsequent figure.*

*Reviewer's responses to the latest revised Ms: Understood your limitations. Yet, such limitations should be more clearly elaborated in the Discussion.*

**Response:** We greatly appreciate the reviewer's suggestion and have clearly stated the limitations in the Discussion (line 400–404).
